# Targeting the Th17/Treg axis: from immunological insights to therapeutic avenues in atherosclerosis

**DOI:** 10.3389/fimmu.2026.1923083

**Published:** 2026-09-10

**Authors:** Zhang Qin, Siqing Chen, Zhe Lyu, Fengyun Zhou, Le Liu, Qi Ai, Zhuo Zeng

**Affiliations:** 1 The Fourth Hospital of Changsha (Changsha Hospital of Hunan Normal University), Changsha, Hunan, China; 2 The First Hospital of Hunan University of Chinese Medicine, Changsha, Hunan, China

**Keywords:** atherosclerosis, chronic vascular inflammation, Th17, therapies, Treg

## Abstract

The paradigm of atherosclerosis has evolved from a passive lipid storage disease to a dynamic immuno-inflammatory disorder, yet the precise immune checkpoints governing its progression remain a therapeutic frontier. This review dissects the yin-yang regulation exerted by CD4^+^ T-cell subsets, with a particular focus on the pro-inflammatory T helper 17 cells (Th17) and the immunosuppressive regulatory T cells (Tregs). We posit that the net balance of the Th17/Treg axis, rather than the isolated activity of either subset, represents an important immunomodulatory determinant that influences plaque fate—operating in concert with other established contributors to atherosclerotic progression, including hemodynamic forces, lipid burden, plaque macrophage content, and coagulation pathways. By integrating recent advances in the metabolic and transcriptional control of this equilibrium, we highlight how its disruption fuels the chronic vascular inflammation central to atherosclerosis. Moreover, we critically evaluate the translational promise of strategically recalibrating this axis, examining next-generation immunotherapies that target lineage-specific pathways to restore homeostasis. This review not only consolidates mechanistic insights but also underscores the urgent clinical potential of harnessing the Th17/Treg balance as a viable strategy to attenuate atherosclerotic disease burden.

## 1 Introduction

Atherosclerosis, the fundamental pathological process underlying ischemic cardiovascular and cerebrovascular diseases such as coronary artery disease, myocardial infarction, and stroke, constitutes a predominant cause of global morbidity and mortality (1–3). Initially conceptualized as a passive lipid deposition disorder, atherosclerosis is now widely recognized as a complex, chronic inflammatory disease driven by metabolic dysfunction (4). The pathogenesis of atherosclerosis involves a multifaceted cascade, including endothelial dysfunction, lipid accumulation, immune cell infiltration, and foam cell formation (4). Both innate and adaptive immune responses are intricately involved in all stages of plaque development and progression (5, 6). Among the diverse immune cells present within atherosclerotic lesions, CD4^+^ T lymphocytes are pivotal players. CD4^+^ T cells differentiate into various subsets with specialized functions, collectively shaping the local inflammatory milieu. Notably, T helper 17 (Th17) cells and regulatory T (Treg) cells have emerged as critical, yet functionally opposing, regulators. Th17 cells, characterized by the production of interleukin-17 (IL-17) and other pro-inflammatory cytokines, promote vascular inflammation, neutrophil recruitment, and plaque instability (7). In contrast, Tregs, a subset of CD4^+^ T cells with potent immunosuppressive capabilities, maintain immune tolerance and homeostasis. They exert their protective effects through cell-contact inhibition and the secretion of anti-inflammatory cytokines such as interleukin-10 (IL-10), transforming growth factor-beta (TGF-β), and interleukin-35 (IL-35), thereby mitigating excessive immune activation (8).

Crucially, the progression, stabilization, or potential regression of atherosclerotic plaques appears to be influenced not merely by the absolute activity of individual cell types, but by the relative balance between Th17 and Treg responses. This shift in the Th17/Treg balance towards a pro-inflammatory state accelerates disease, while biased immunomodulation provides protection (9). Therefore, the Th17/Treg axis represents an important immunological checkpoint in atherosclerosis. This review aims to synthesize current knowledge on the distinct roles of Th17 and Treg cells in atherosclerosis, elucidate the mechanisms governing their delicate balance, and discuss how its dysregulation contributes to disease pathogenesis. Furthermore, we will explore the promising translational potential of targeting this axis, highlighting emerging therapeutic strategies designed to restore immunological equilibrium and combat atherosclerosis.

## 2 Functional specialization and plasticity of the Th17/Treg axis

### 2.1 Functional specialization of Th17 cells: orchestration of pro-inflammatory responses

Th17 cells, as a distinct subset of CD4^+^ T helper cells, play a critical pro-inflammatory and tissue-defensive role in adaptive immune responses (10, 11). Their cellular fate and function are primarily governed by the lineage-specific transcription factor Retinoic acid-related orphan receptor gamma t (RORγt) (12). The expression of this factor drives the production of their signature cytokine—IL-17A—which is also the origin of their nomenclature (13).

The differentiation of Th17 cells is a tightly regulated process that depends on a precise combination of local cytokines. The canonical differentiation pathway is initiated by the synergy of TGF-β and interleukin-6 (IL-6), while interleukin-21 (IL-21) can exert a similar signal-amplifying function in this process (14, 15). Notably, the concentration of TGF-β plays a decisive, switch-like role: low concentrations of TGF-β cooperate with IL-6/IL-21 to promote stable Th17 differentiation by upregulating IL-23 receptor expression; conversely, high concentrations of TGF-β suppress the Th17 phenotype and instead promote differentiation toward Tregs by inducing the expression of forkhead box P3 (Foxp3) and inhibiting the activity of RORγt (16). Although interleukin-23 (IL-23) is not essential for initial differentiation, it is crucial for the maintenance, expansion, and conversion of Th17 cells into highly inflammatory subsets with pathogenic functions (17).

Under pathological conditions, the function of Th17 cells shifts from defense to attack, making them core effector cells driving various chronic inflammatory and autoimmune diseases (18, 19). Their pathogenicity is manifested in two aspects. First, there is the secretion of a broad spectrum of pro-inflammatory mediators. Activated Th17 cells can produce a characteristic cytokine profile in large quantities, including IL-17A, IL-17F, IL-21, IL-22, granulocyte-macrophage colony-stimulating factor (GM-CSF), and tumor necrosis factor-α (TNF-α) (20, 21). Second, these cytokines initiate and amplify tissue inflammation through complex network effects. For example, IL-17 can activate endothelial cells, epithelial cells, and fibroblasts, inducing the secretion of chemokines and pro-inflammatory factors (such as IL-6 and TNF-α), thereby recruiting myeloid cells like neutrophils (22, 23); GM-CSF can promote the activation and survival of monocytes/macrophages (24, 25). This cascade of reactions collectively leads to immunopathological tissue damage (26). Precisely because of this, Th17 cells and their effector pathways have been confirmed to play key roles in the pathogenesis of various chronic inflammatory diseases, including atherosclerosis (27), myocardial infarction (28), rheumatoid arthritis (29), and autoimmune myocarditis (30).

### 2.2 Functional specialization of Treg cells: maintenance of immune tolerance

Tregs are a core subset that maintains the body’s immune homeostasis (31). Through their potent immunosuppressive functions, they play a key regulatory role in various inflammatory diseases, including atherosclerosis (32). Tregs are mainly divided into two categories: natural Tregs that develop in the thymus, and induced Tregs that are generated in peripheral sites from naïve T cells under specific cytokine environments, such as TGF-β (33, 34). Both types of cells highly express the transcription factor Foxp3, which acts as the “master switch” for Treg cell lineage specification and functional execution (35). The expression of Foxp3 not only determines the immunosuppressive phenotype of Treg cells but also allows them to adapt to the inflammatory microenvironment through global regulation of gene expression programs (36). Research indicates that Foxp3 can reprogram T cell metabolic pathways, enabling them to preferentially utilize fatty acid oxidation(FAO) rather than glycolysis for energy acquisition; this unique metabolic reprogramming allows Treg cells to survive and function in inflammatory microenvironments characterized by high lactate and low glucose levels, such as atherosclerotic plaques, forming a metabolic competition with Th17 cells, which primarily rely on glycolysis for energy, thereby achieving immunosuppression at a deeper level (37, 38). Treg cells primarily exert their immunoregulatory functions through three mechanisms: (1) contact-dependent inhibition, for example, by the high surface expression of cytotoxic T-lymphocyte-associated protein 4 (CTLA-4) binding to CD80/CD86 on antigen-presenting cells, transmitting an inhibitory signal; (2) secretion of inhibitory cytokines, mainly including IL-10, TGF-β, and IL-35 (39–41). These factors broadly suppress the activation and function of effector T cells, dendritic cells, and macrophages in a paracrine manner (42, 43); (3) competitive consumption of interleukin-2 (IL-2) in the microenvironment, thereby depriving effector T cells of this key growth factor essential for their growth (44, 45). In atherosclerosis, Treg cells, through the aforementioned mechanisms, particularly the secretion of IL-10 and TGF-β, can effectively suppress the overactivation of other immune cells within plaques, reduce vascular wall inflammation, stabilize plaques, and inhibit their progression (46, 47). It is particularly important to note that there is a multifaceted and complex relationship between Treg cells and Th17 cells in terms of differentiation, function, and fate (48). TGF-β is a key inducing factor for Treg cell differentiation (16). In its presence, the types of cytokines in the microenvironment determine the fate: the addition of pro-inflammatory factors such as IL-6 drives naïve T cells to differentiate into Th17 cells, whereas the absence of such factors leads to Treg differentiation. Thus, they share the starting point of the TGF-β signaling pathway but subsequently develop into functionally antagonistic subsets (49, 50). Notably, the expression of the Th17-specific transcription factor RORγt has been reported within a subset of Treg cells (51). This phenomenon, which seems contradictory to their role in opposing Th17 cells, is not necessarily paradoxical. It may be attributed to flexible epigenetic programming changes in T cells and the retention of Th programs acquired before conversion in Treg cells, although the specific mechanisms require further investigation (49). The functional characteristics and plasticity of Th17 and Treg are discussed in the next section. The ratio of Treg/Th17 and the balance of their functional states are critical nodes determining whether the immune response tilts towards promoting or suppressing inflammation (52, 53). This provides a crucial targeting rationale for intervening in diseases like atherosclerosis.

### 2.3 Functional plasticity and lineage instability of Th17 and Treg cells

The developmental trajectories of Th17 and Treg cells are not strictly fixed; rather, these subsets exhibit considerable phenotypic flexibility in response to shifting microenvironmental signals (54). This phenomenon of lineage plasticity has emerged as an important determinant of immune homeostasis, with particular relevance to chronic inflammatory conditions such as atherosclerosis, where the balance between pro-inflammatory Th17 cells and immunosuppressive Tregs critically influences disease progression and plaque stability (55, 56). Within the arterial wall, where inflammatory cues fluctuate throughout the course of lesion development, the dynamic phenotypic adaptation of these compartments adds a layer of complexity that static measurements of subset frequencies fail to capture (55, 57, 58). An additional layer of complexity arises from the inherent heterogeneity of the Treg compartment, as not all Tregs exhibit equivalent susceptibility to lineage instability (59). Thymic-derived Tregs and peripherally induced Tregs differ in their epigenetic landscapes, with peripherally induced Tregs generally exhibiting less complete demethylation of the Treg-specific demethylated region (TSDR) and consequently greater plasticity under inflammatory conditions (60–62). Furthermore, Tregs can be sub-stratified based on surface markers such as CD45RA, which distinguishes naïve from antigen-experienced subsets, with the latter displaying heightened susceptibility to phenotypic switching under inflammatory pressure (63, 64). This functional heterogeneity has direct implications for atherosclerosis, where plaque-infiltrating Tregs may exhibit varying degrees of stability depending on their origin, activation history, and local microenvironmental cues (65–67).

**Figure 1:**
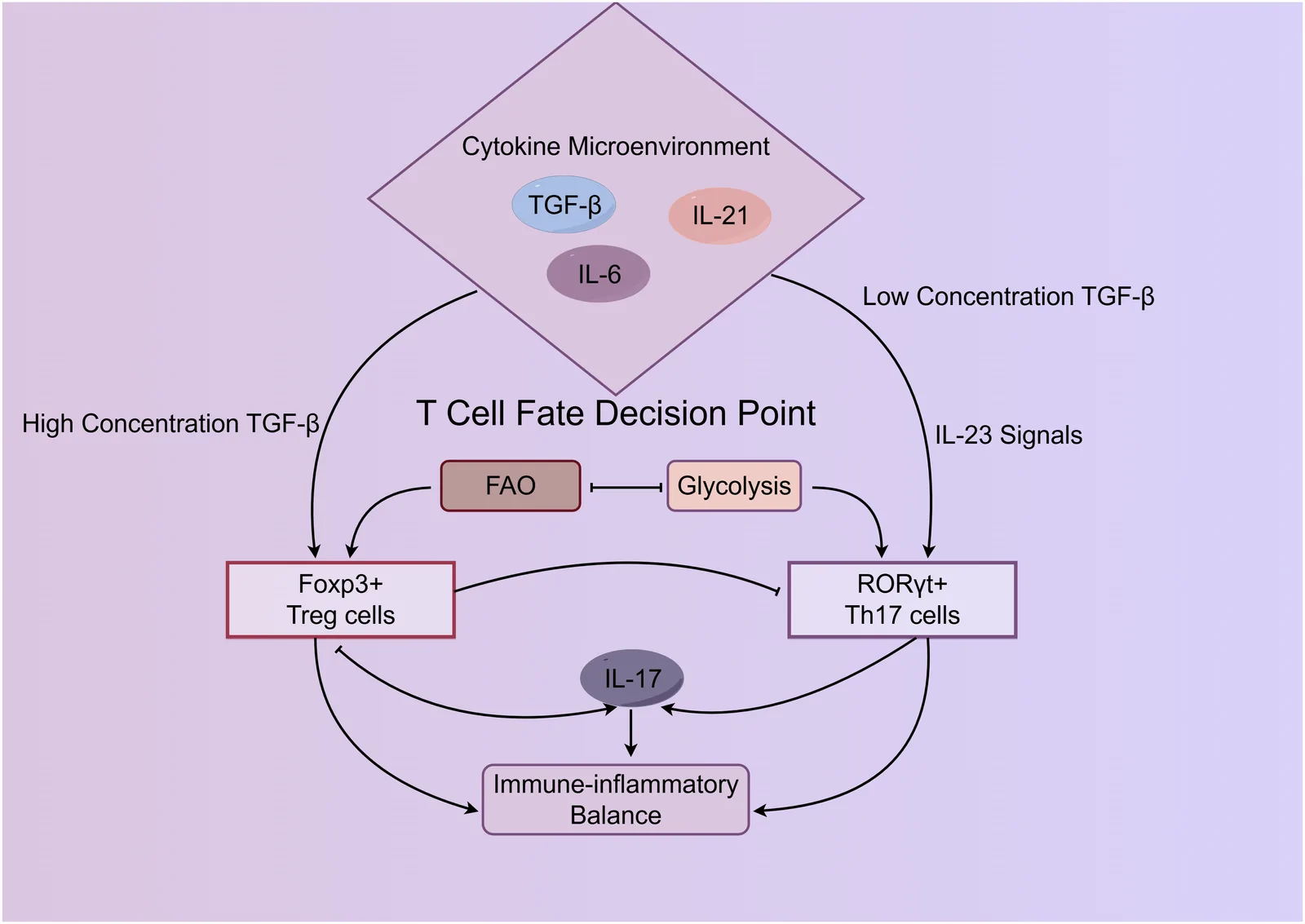
Cytokine and metabolic regulation of Th17/Treg balance. The fate decision between Th17 and Treg differentiation is governed by TGF-β concentration: low-dose TGF-β combined with IL-6 and IL-21 activates IL-23 signaling to drive Th17 commitment via RORγt, whereas high-dose TGF-β upregulates Foxp3 and suppresses RORγt to promote Treg differentiation. Metabolically, glycolysis favors Th17 polarization, whereas FAO supports Treg development. Notably, IL-17, a key RORγt-driven Th17 effector, is counteracted by Foxp3 overexpression, which also curbs Th17 differentiation; conversely, IL-17 suppresses Foxp3 signaling (By Figdraw).

The cytokine milieu plays a critical role in modulating the relationship between Treg and Th17 cells. Beyond the classical upstream signals—IL-6 and TGF-β—addressed in the preceding section, the downstream effector cytokine IL-17 itself constitutes another key signaling molecule that influences the balance between these two functionally antagonistic subsets. As a canonical Th17-derived cytokine governed by RORγt, IL-17 serves as an important mediator of inflammatory responses (12). Overexpression of Foxp3 has been demonstrated to abrogate both the inflammation-induced upsurge of IL-17 and the differentiation of Th17 cells (68). In parallel, IL-17 negatively modulates Foxp3 signaling, thereby establishing a reciprocal inhibitory crosstalk. Of note, the loss of Foxp3, the lineage-defining transcription factor for Tregs, destabilizes their regulatory identity and promotes their conversion into exTregs that have acquired a pro-inflammatory T-cell program, a process that may be favored by the hypoxic microenvironment characteristic of atherosclerotic plaques (67). This RORγt/Foxp3 bidirectional antagonism represents a pivotal regulatory module governing the inflammatory response (69). In the context of atherosclerosis, the inflammatory milieu within the plaque—characterized by oxidative stress, lipid accumulation, and persistent cytokine production—actively promotes Treg destabilization and Th17 differentiation, contributing to the persistence of vascular inflammation and the progression toward plaque vulnerability (70)(**Figure 1**). Consequently, strategies aimed at preserving Treg identity and constraining Th17 responses represent a promising area of therapeutic investigation.

### 2.4 Spatial heterogeneity and redefinition of T-Cell subsets in the atherosclerotic plaque

The phenotypic and functional characterization of Th17 and Treg cells in atherosclerosis has traditionally relied upon analyses of large cell populations isolated from peripheral blood or digested whole aortas (9, 71).

While these approaches have established the importance of the Th17/Treg balance in atherosclerosis, they average molecular signals across thousands of cells and fail to preserve the spatial context in which T cells operate within the complex architecture of the plaque. Recent advances in single-cell and spatial transcriptomic technologies have begun to overcome these limitations, revealing an unexpected degree of T-cell heterogeneity and spatial organization that challenges conventional subset classifications (72, 73). These technologies offer a unique opportunity to dissect not only the individual phenotypes of Th17 and Treg cells but also the spatial and clonal relationships that govern their functional dynamics within the plaque microenvironment.

Unbiased transcriptional profiling of plaque-infiltrating T cells has identified multiple CD4^+^ T cell clusters that extend beyond the classical Th1/Th17/Treg paradigm (74–76). Single-cell RNA sequencing of C-C chemokine receptor 5 (CCR5)^+^ CD4^+^ T cells isolated from atherosclerotic Apoe^-^/^-^ mice identified a plastic IFNγ^+^Foxp3^+^ subset—Th1/Tregs—that originates from genuine Tregs and exhibits a distinct transcriptional landscape marked by the simultaneous expression of Treg- and Th1-associated genes (47). Critically, this Th1/Treg population represents an intermediate state bridging the regulatory and pro-inflammatory arms of the CD4^+^ T cell compartment (47). Extending this concept to the Th17/Treg axis, transcriptional analyses of Tregs isolated from dyslipidemic individuals have revealed a mixed Th1/Th17/Treg profile, with elevated expression of IL-17, RORγt and IFNγ alongside Foxp3 and IL-10 (77). This observation suggests that, analogous to Th1/Tregs, cells co-expressing Th17- and Treg-associated genes may represent transitional states that can adopt either regulatory or pro-inflammatory effector functions depending on local microenvironmental cues.

Recent evidence reveals that Tregs exhibit phenotypic and functional reprogramming toward a proinflammatory profile as early as the initial stages of atherosclerosis, accompanied by their increased accumulation at atherosclerotic lesions—a process critically governed by CCR5-mediated T-cell trafficking—which collectively underscores the spatial heterogeneity of T cells within atheromatous plaques and their contributory role in disease progression (78). In human atherosclerotic plaques, CCR5^+^Foxp3^+^ cells have been identified within the lesions, indicating that Tregs expressing this chemokine receptor are present at the site of disease and are therefore positioned to interact with locally produced inflammatory signals that may drive their conversion toward a Th17-like phenotype (78). Furthermore, in the aortas of atherosclerotic mice, CCR5^+^CD4^+^ T cells are enriched, and the vast majority of these cells lack C-X-C motif chemokine receptor 3 (CXCR3) while retaining CCR5 expression (47). This characteristic receptor profile points to a specialized homing behavior that may preferentially direct these plastic T cells toward plaque subregions enriched in Th17-polarizing cues, including IL-6, IL-1β, and hypoxic conditions (79, 80). This spatial compartmentalization is particularly relevant to Th17/Treg biology: if Th17-polarizing cytokines are enriched in certain plaque regions, such as the fibrous cap shoulder or the necrotic core border, then Tregs that home to these areas may be more susceptible to destabilization and conversion toward a Th17-like state. Conversely, regions characterized by TGF-β predominance may favor Treg retention and stability. Thus, the spatial organization of the plaque creates a mosaic of local microenvironments, each with distinct Th17 versus Treg promoting potential, and the net balance across these regions determines the overall disease trajectory.

Elucidating the spatial coordinates of Th17/Treg plasticity will be instrumental in devising spatially informed immunomodulatory approaches that preserve Treg functionality within regions where regulatory activity is most critically required, while simultaneously constraining Th17 differentiation in inflammatory hotspots. As will be discussed in the subsequent sections, emerging evidence implicates epigenetic regulation, metabolic reprogramming, microbial metabolites, and protein signaling pathways as key mechanisms that modulate Th17/Treg balance. Stabilization of lineage identity through these pathways offers potential avenues for preserving the regulatory compartment and alleviating the inflammatory burden of atherosclerotic disease.

## 3 The Janus face of immunity: the dualistic role of the Th17/Treg axis in atherosclerosis pathogenesis and protection

### 3.1 The perilous imbalance: a pro-inflammatory shift towards Th17 dominance in atherosclerosis

The pathogenesis of atherosclerosis is intrinsically linked to a marked dysregulation of the adaptive immune system, characterized by a decisive skewing of the Th17/Treg balance towards a pro-inflammatory Th17 phenotype. This imbalance is not a passive accompaniment but an active driver that sustains and amplifies the chronic inflammatory state within the arterial wall, critically influencing plaque initiation, progression, and ultimate stability (81, 82).

The atherosclerotic microenvironment itself is a potent architect of this immune deviation. An abundance of local factors, including oxidized low-density lipoprotein (ox-LDL) and a milieu rich in specific cytokines, creates conditions that favor Th17 commitment and suppress Treg functionality (83). A key driver is the synergy between IL-6 and TGF-β, which steers naïve CD4+ T cells towards the Th17 lineage, a process further potentiated by IL-21 (84, 85). This Th17-polarizing environment is often coupled with a relative deficiency in signals crucial for Treg homeostasis. For instance, adequate IL-2 signaling is vital for Treg survival and suppressive function, yet its availability may be limited in inflamed tissues (49). Consequently, atherosclerotic plaques consistently demonstrate an increased frequency and heightened activity of Th17 cells, frequently juxtaposed with a numerical or functional impairment of Treg cells (55, 86). The direct consequence of this Th17 bias is the enhanced local production of its hallmark effector cytokine, IL-17A (87). While IL-17A can be produced by various innate and adaptive immune cells, its overproduction within the plaque is a central mediator of vascular damage (88). IL-17A exerts its effects by binding to a receptor complex containing IL-17 receptor A (IL-17RA), which is constitutively expressed on key vascular cells such as endothelial cells (ECs) and vascular smooth muscle cells (VSMCs) (11, 89). This binding initiates a destructive signaling cascade: ECs are activated to upregulate adhesion molecules (e.g., VCAM-1) and secrete potent chemokines like C-X-C motif chemokine ligand-1 (CXCL1) and C-X-C motif chemokine ligand-8 (CXCL8), thereby orchestrating the sustained recruitment of monocytes and neutrophils into the subendothelial space (90, 91). Within the developing plaque, IL-17A acts on macrophages and VSMCs, stimulating them to produce additional pro-inflammatory cytokines (e.g., IL-1β, IL-6, TNF-α), matrix-degrading enzymes such as matrix metalloproteinases (MMPs), and reactive oxygen species (ROS) (90, 92). This creates a self-amplifying inflammatory loop, as these newly produced mediators further promote immune cell infiltration and activation (93, 94). The particular potency of IL-17A stems from its ability to synergize powerfully with other cytokines like TNF-α, leading to an exponential increase in inflammatory gene expression and tissue damage beyond what either cytokine could achieve alone (95, 96). Simultaneously, the relative insufficiency of Treg-mediated suppression fails to provide an adequate counterbalance to this inflammatory onslaught. Tregs normally maintain immune quiescence through multiple mechanisms, including the secretion of anti-inflammatory cytokines like IL-10 and TGF-β, and via direct cell-contact inhibition (97, 98). Their impaired function or reduced presence in the atherosclerotic lesion results in a loss of this crucial “braking” signal (32). Consequently, the activation of effector T cells and macrophages proceeds unchecked, foam cell formation is exacerbated, and pathways leading to inflammation resolution are fundamentally undermined (99, 100). Strong preclinical evidence solidifies the causal role of this skewed axis. In apolipoprotein E-deficient (ApoE-/-) mouse models, a diet-induced atherogenic state is associated with a marked upregulation of IL-17 expression in aortic tissue, coinciding with accelerated plaque formation (101, 102). Moreover, atherosclerotic plaques in these models show a clear enrichment of Th17 cells compared to plaque-free arterial walls while administration of recombinant IL-17A accelerates disease progression (79, 103). Importantly, therapeutic neutralization of IL-17A with monoclonal antibodies not only limits plaque growth but also promotes features associated with stability, such as thicker fibrous caps, increased collagen deposition, and reduced apoptotic cell burden (88, 104).

Translational research in humans corroborates these experimental findings. IL-17A is reliably detected within human carotid and coronary atherosclerotic plaques retrieved during endarterectomy (105). Clinically, its presence carries significant prognostic weight; higher levels of IL-17A are found in plaques from symptomatic patients and correlate with histological markers of plaque vulnerability (105, 106). Furthermore, circulating levels of IL-17 have been associated with a greater burden of high-risk coronary plaque features on imaging studies, independent of total plaque volume (106). Emerging evidence also extends the pro-atherogenic role of this axis to include pro-thrombotic effects, with IL-17 shown to enhance platelet activation—a critical step in acute coronary syndrome pathogenesis (107, 108). Despite this considerable body of evidence, a note of complexity exists.

The pathogenic paradigm of IL-17A in atherosclerosis, while supported by the preponderance of experimental data, is not without substantive dissent. In a particularly illustrative series of experiments, IL-17A deficiency in the ApoE^-^/^-^ background was achieved through germline compound knockout and evaluated across three independent atherosclerosis induction models, including diet-induced hyperlipidemia, angiotensin II-mediated hypertension, and hemodynamic perturbation via partial carotid ligation; this genetic ablation conferred multiple favorable metabolic and vascular phenotypes under high-fat feeding, including diminished systemic interferon-gamma (IFN-γ) production, reduced aortic superoxide production, increased aortic nitric oxide levels, and attenuated leukocyte and dendritic cell infiltration into the arterial wall (109). Paradoxically, however, these salutary inflammatory and redox modifications did not translate into measurable reductions in aortic plaque burden following either high-fat diet or angiotensin II infusion. In the partial carotid ligation model, IL-17A deficiency did not affect the percentage of stenosis but unexpectedly promoted adverse outward remodeling, suggesting that the cytokine may exert divergent effects on plaque geometry and arterial wall architecture. Furthermore, neutralization of the related isoform IL-17F in IL-17A/ApoE^-^/^-^ mice did not alter atherosclerotic outcomes in this model, and circulating IL-17A levels showed no correlation with carotid intima-media thickness in a human cohort, collectively indicating that neither functional redundancy within the IL-17 family nor systemic IL-17A concentrations account for the observed disconnect between inflammatory modulation and structural atherosclerotic burden (109). These discordant observations point to several methodological sources of variability that likely underlie these discrepant outcomes. The timing of cytokine ablation relative to plaque initiation versus progression represents one critical variable. The anatomical site of lesion assessment constitutes another source of divergence, given that regional hemodynamic differences across the aortic arch, aortic root, and thoracoabdominal aorta may influence lesion characteristics and responsiveness to immunomodulation. The completeness of pathway disruption, whether through genetic deletion, pharmacological neutralization, or receptor antagonism, has also been implicated as a source of discordant findings. Furthermore, developmental compensation in constitutive knockout models may not faithfully recapitulate the effects of acute therapeutic blockade in an established disease setting. These variables collectively underscore that the net impact of IL-17A on atherogenesis is exquisitely context-dependent, a conclusion that carries significant implications for therapeutic translation. This experimental uncertainty is further contextualized by the clinical experience with anti-IL-17A biologics currently approved for autoimmune indications such as psoriasis, psoriatic arthritis, and ankylosing spondylitis (110–112). Agents including secukinumab and ixekizumab have demonstrated efficacy in managing these immune-mediated inflammatory conditions, yet their cardiovascular profile remains incompletely defined. This evidentiary gap does not diminish the pathogenic relevance of IL-17A in atherosclerosis, a cytokine whose pro-inflammatory effector functions in endothelial activation, leukocyte recruitment, and plaque destabilization have been extensively documented in the mechanistic literature reviewed above. Rather, the disconnect between substantial preclinical mechanistic support and the absence of clinical cardiovascular outcome data with IL-17A inhibitors highlights the inherent challenges in translating single-target immunomodulation from experimental models to human disease.

Several interpretive frameworks may reconcile this apparent paradox while preserving the mechanistic centrality of the Th17/IL-17A pathway. One possibility is that the atheroprotective dividend of Th17/Treg modulation depends on integrated regulation of multiple downstream effectors, encompassing not only IL-17A but also IL-17F, IL-22, and granulocyte-macrophage colony-stimulating factor. Under this framework, isolated neutralization of IL-17A alone would be insufficient to achieve meaningful cardiovascular risk modification. This interpretation finds support in the observation that IL-17F neutralization failed to alter atherosclerosis in IL-17A-deficient mice, suggesting that more comprehensive or alternative targeting strategies may be required (109). Alternatively, the timing of intervention may be critical, as IL-17A may promote early lesion initiation while its role in established disease could be more complex or even subject to compensatory mechanisms that render late-stage blockade less effective. A third consideration is that the Th17/Treg axis influences cardiovascular outcomes through mechanisms extending beyond IL-17A itself, including Treg-mediated suppression of other pathogenic T-cell subsets, indirect effects on lipid metabolism, and modulation of endothelial function that are not captured by IL-17A-directed therapies alone. Despite these complexities and the absence of positive cardiovascular outcome data for IL-17A inhibitors, the mechanistic evidence continues to firmly position the Th17/Treg disequilibrium as a central pathological driver in atherosclerosis. The clinical null results with anti-IL-17A monotherapy should be interpreted not as evidence against the Th17/IL-17A axis, but rather as a critical reminder that the relationship between a mechanistically central pathway and its therapeutic modulation is rarely linear. The context-dependent nature of IL-17A biology, the functional redundancy within the IL-17 family, and the likelihood that optimal immunomodulation requires multifaceted rather than single-target approaches all point to the same conclusion: the translational journey from mechanistic insight to clinical application in this field will demand continued refinement of our understanding of when, how, and in whom to intervene. These unresolved questions, far from undermining the Th17/Treg paradigm, define the frontier of ongoing investigation and highlight the imperative for future studies designed to dissect the stage-specific and context-dependent contributions of IL-17A to human atherosclerotic disease.

### 3.2 The protective pivot: atheroprotective mechanisms of Treg-biased immune regulation

In contrast to the pathogenic consequences of a Th17-dominant state, a substantial body of evidence underscores the atheroprotective potential of skewing the Th17/Treg balance towards Treg dominance. This shift is not merely a reduction in inflammation but an active engagement of multifaceted immunosuppressive and tissue-stabilizing programs that can halt disease progression and even induce plaque regression.

Treg cells, defined by the expression of the master transcription factor Foxp3, constitute a critical subset of CD4^+^ T cells dedicated to maintaining immune homeostasis (113). Their protective role in atherosclerosis is executed through a diverse array of mechanisms. A primary mode of action is the secretion of anti-inflammatory cytokines, including IL-10, TGF-β, and IL-35 (41, 114). IL-10 and TGF-β potently suppress the activation and function of pro-inflammatory effector T cells like Th1 and Th17 cells, and also modulate macrophage phenotype, favoring the anti-inflammatory M2 state over the pro-inflammatory M1 state, thereby reducing foam cell formation (115–117). IL-35 is induced by pro-inflammatory stimuli and has the ability to convert naive T cells into new subsets of regulatory cells. It plays a role in the early progression of atherosclerosis by inhibiting the activation of endothelial cells, which helps prevent cardiovascular inflammatory responses (118). One study demonstrated that administering exogenous IL-35 alleviated atherosclerosis in Apoe-/- mice; While IL-35-deficient mice exhibited reduced suppressive function in Treg cells, indicating that IL-35 is involved in regulating Treg cells and has protective effects against atherosclerosis (119). Concurrently, Treg cells employ direct cell-contact inhibition via surface molecules such as cytotoxic T-lymphocyte-associated antigen 4 (CTLA-4) and lymphocyte activation gene-3 (LAG-3) (120, 121). CTLA-4, by binding to CD80/CD86 on antigen-presenting cell(APC) with higher affinity than the co-stimulatory receptor CD28, effectively dampens the signals required for effector T cell activation (122, 123). LAG-3 can bind to MHC class II molecules on the surface of APC and inhibit its immune function and reduce inflammation through the tyrosine-activating motif pathway of immune receptors (124). Furthermore, Treg cells express high levels of the high-affinity IL-2 receptor α chain (CD25) (125). By competitively sequestering IL-2 in the local microenvironment, they restrict the availability of this critical growth factor for proliferating effector T cells, a mechanism known as “cytokine deprivation” (126). The atheroprotective significance of Treg cells is strongly supported by clinical and preclinical correlations (127, 128). In patients with atherosclerosis, the frequency and suppressive function of circulating Treg cells are often diminished, and this reduction correlates with disease severity and plaque instability (129, 130). A cohort study involving 700 patients demonstrated that low levels of circulating CD4^+^FoxP3^+^ T cells were significantly associated with an increased risk of myocardial infarction (131). Additional studies have reported that Treg ratios are reduced in patients with myocardial infarction compared with those with stable angina (132), and that lower Treg/CD4^+^ T-cell ratios may predict higher cardiovascular event rates (133, 134). Collectively, these findings reinforce the translational relevance of Treg-mediated immunoregulation in atherosclerosis, although the precise predictive value of Treg frequency across different clinical manifestations warrants further investigation.

Conversely, experimental interventions that augment Treg cells yield consistent therapeutic benefits. In ApoE^-^/^-^ mouse models, genetic or antibody-mediated depletion of Treg cells exacerbates atherosclerotic lesion size and instability (135). Interestingly, the adoptive transfer of *in vitro*-expanded Treg cells into atherosclerotic mice significantly reduces plaque burden, increases collagen content and smooth muscle cell presence in the fibrous cap, and downregulates the expression of MMPs like MMP-2 and MMP-9, collectively promoting a stable plaque phenotype (136, 137). Beyond direct suppression, Treg cells influence the atherosclerotic milieu through metabolic and chemokine-mediated crosstalk. Emerging research highlights the role of the CD39-CD73-adenosine pathway. Treg cells expressing the ectonucleotidases CD39 and CD73 can hydrolyze pro-inflammatory extracellular ATP into immunosuppressive adenosine, thereby dampening local inflammation (138, 139). The CCL1-CCR8 chemokine axis has also been identified as crucial for Treg function within plaques (140). Disruption of this axis impairs Treg-mediated suppression and accelerates atherosclerosis in mice, whereas its engagement enhances Treg regulatory capacity (141). Pharmacological agents like statins and aspirin have also been shown to exert part of their beneficial effects by favorably modulating the Treg/Th17 balance and enhancing Treg function via pathways such as the CD39-adenosine axis (139).

**Figure 2:**
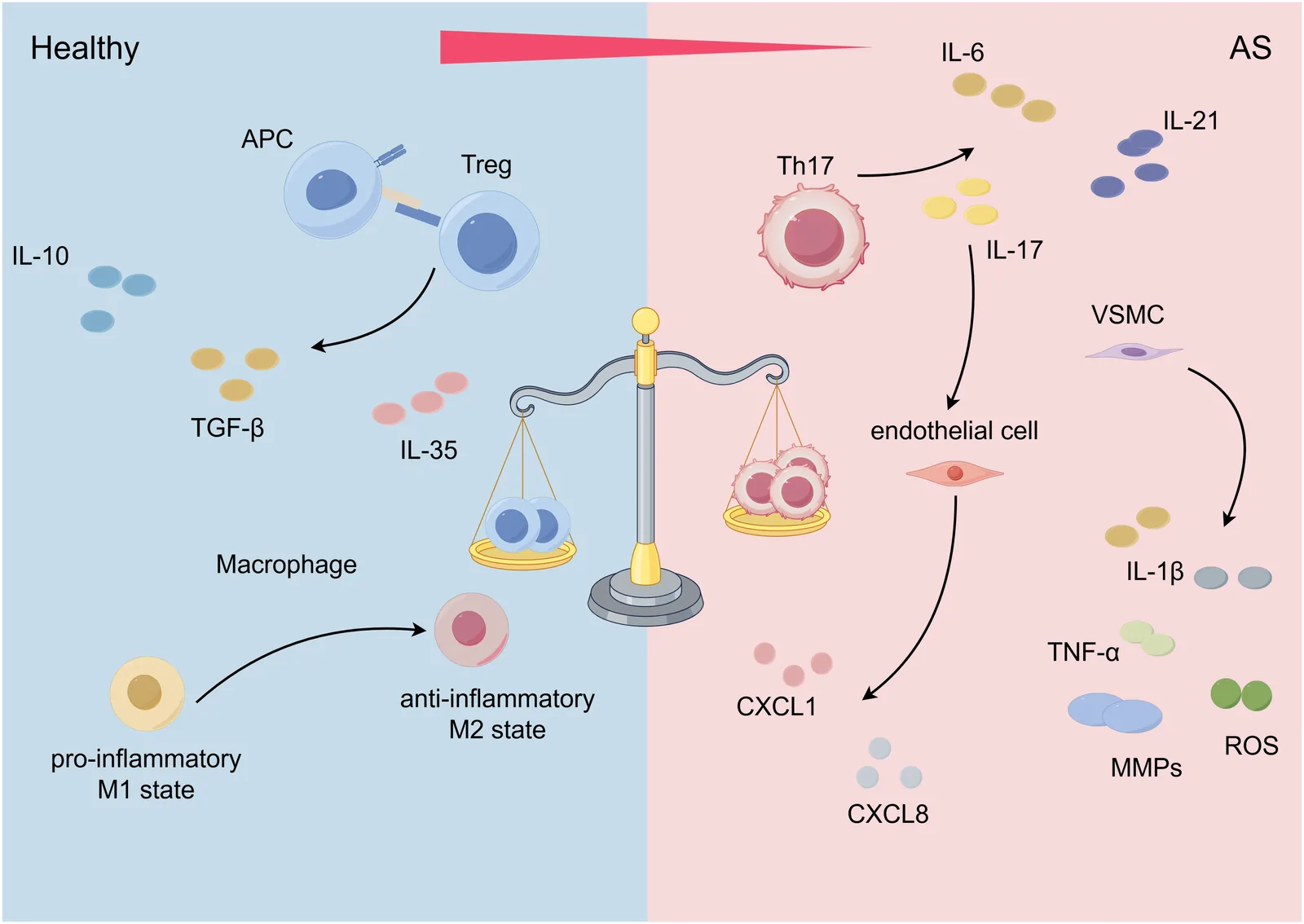
An imbalance between Th17 and Treg cells can lead to the development of atherosclerosis. Tregs interact with antigen-presenting cells to produce anti-inflammatory cytokines and help promote a healthier M2 macrophage state, which lowers foam cell formation. In contrast, Th17 cells release pro-inflammatory cytokines like IL-17 that affect key blood vessel cells, triggering a harmful inflammatory response. This process generates more pro-inflammatory signals and enzymes, creating a cycle that encourages further immune cell activation and inflammation (By Figdraw).

In summary, reinforcing the Treg arm of the immune system represents a powerful and clinically viable strategy to restore immunological balance, suppress vascular inflammation, and stabilize atherosclerotic plaques, thereby translating deep immunological insights into tangible therapeutic avenues for cardiovascular disease. Therefore, therapeutic strategies aimed at rebalancing this axis—by suppressing the Th17 pathway, augmenting Treg function, or a combination of both—represent a rational and highly promising frontier in the search for novel immunomodulatory treatments for atherosclerosis (**Figure 2**).

## 4 Regulation and operational mechanisms of Th17/Treg balance in atherosclerosis

### 4.1 Epigenetic regulation of the Th17/Treg axis in atherosclerosis: mechanisms and therapeutic implications

The dynamic balance between pro-inflammatory Th17 cells and anti-inflammatory Treg cells constitutes a central immunological determinant in atherosclerosis pathogenesis (142, 143). This equilibrium is governed by epigenetic mechanisms—reversible, heritable modifications that regulate gene expression without altering the DNA sequence (144, 145). Key epigenetic processes, including histone modifications, DNA methylation, and associated enzymatic activities, critically direct T cell lineage commitment, functional plasticity, and inflammatory potential (146). Dysregulation of this epigenetic landscape skews the Th17/Treg axis toward a pro-inflammatory state, thereby accelerating plaque development and instability (147). As the catalytic subunit of Polycomb Repressive Complex 2 (PRC2), Enhancer of Zeste Homolog 2 (EZH2) deposits the repressive histone mark trimethylation of histone H3 at lysine 27 (H3K27me3) (148). In human advanced atherosclerotic plaques, EZH2 expression is markedly elevated and predominantly localized to the nuclei of infiltrating T lymphocytes (149). The functional significance of this observation is underscored by studies in apolipoprotein E-deficient mice (ApoE−/−) mice with T cell-specific EZH2 deletion. Genetic ablation of EZH2 specifically in CD4+ T cells, but not in CD8+ T cells, confers atheroprotection, characterized by reduced lesion size, increased plaque collagen content, and decreased macrophage accumulation. Mechanistically, loss of EZH2 in CD4+ T cells reprograms their phenotype toward a type 2 immune response (149). This is evidenced by upregulated interleukin-4 (IL-4) expression, expansion of Th2 effector cells, and a significant increase in a specific subset of invariant Natural Killer T (iNKT) cells characterized by high expression of the transcription factor Promyelocytic Leukemia Zinc Finger (PLZF) (149). These PLZF^+^ iNKT cells, referred to as iNKT2 cells, are known to promote a type 2 immune response (149, 150). Moreover, the reduction in EZH2 promoted the differentiation of Treg cells inCD4+ cells (151). Therefore, the inhibition of EZH2 in CD4^+^T cells typically promotes the immune regulatory program that protects arteriosclerosis (149, 152). In parallel, the stability and function of the Treg compartment are exquisitely sensitive to DNA methylation patterns (153). The expression of Foxp3, the master transcription factor defining Treg identity, is controlled by the methylation status of a conserved regulatory element within its locus, the TSDR (154). Hypermethylation of the TSDR, maintained by DNA methyltransferase 1 (Dnmt1), suppresses Foxp3 transcription (155, 156). Pharmacological inhibition of DNA methylation with 5-azacytidine (Aza) potently induces TSDR demethylation, upregulates Foxp3 expression, and drives the conversion of naïve CD4^+^ T cells into functional induced Tregs *in vitro (*157, 158).The therapeutic potential of this pathway is illustrated by adoptive transfer experiments showing that the infusion of Azacitidine-generated Tregs into atherosclerotic ApoE-/- mice expands the peripheral Treg population, suppresses systemic inflammation, and reduces plaque burden, while the overexpression of Dnmt1 in CD4^+^ T cells negates the Aza-induced expression of Foxp3 (147). These findings establish DNA methylation, specifically Dnmt1-mediated repression of the Foxp3 locus, as a critical barrier to Treg differentiation in atherosclerosis and a viable target for immune modulation (147, 155). Beyond methylation, post-translational acetylation mechanisms are essential for Treg functionality (159). The histone acetyltransferase P300/CBP-associated factor (PCAF) directly acetylates the Foxp3 protein, a modification crucial for enhancing its transcriptional activity and protein stability (160). *In vivo* studies in ApoE-/- mice with PCAF deficiency reveal a direct link between this epigenetic modifier and atherosclerosis:PCAF-deficient mice exhibit a significant reduction in circulating Foxp3^+^ Tregs and, despite having comparable levels of hypercholesterolemia and systemic inflammatory cytokines like TNF-α and IL-6, they develop much larger atherosclerotic lesions than control mice, indicating that the exacerbation of the disease is primarily driven by the loss of Treg-mediated immunoregulation, which underscores the essential role of PCAF-mediated acetylation in maintaining a functional Treg compartment capable of controlling atherosclerotic inflammation (161).

Collectively, these findings position epigenetic regulation as a central control layer for the Th17/Treg axis in atherosclerosis. The coordinated actions of EZH2 (via H3K27me3), Dnmt1 (via DNA methylation), and PCAF (via acetylation) establish a dynamic network that can be perturbed by the disease microenvironment, leading to immune imbalance and the inherent reversibility of epigenetic marks presents a promising therapeutic opportunity (162, 163). Targeted pharmacological agents, such as small-molecule inhibitors against EZH2 or Dnmt1, or activators of specific acetyltransferases, offer promising strategies to therapeutically recalibrate the Th17/Treg equilibrium (164–166). By promoting a shift toward Treg-me diated tolerance and suppressing pro-inflammatory Th17 responses, the epigenetic interventions hold significant potential to mitigate plaque inflammation, enhance stability, and ultimately alter the progression of atherosclerosis (167–169).

### 4.2 Immunometabolic reprogramming in Th17/Treg dynamics: a key determinant in atherosclerosis pathogenesis

The functional polarization and stability of Th17 and Treg cells in atherosclerosis are governed by a multi-layered regulatory framework, with cellular metabolic reprogramming representing a critical operational tier (170). The atherosclerotic plaque microenvironment, characterized by hypoxia, nutrient flux alterations, and bioactive lipid accumulation, imposes specific metabolic demands on infiltrating immune cells (171). The divergent metabolic commitments of CD4^+^ T cell subsets—specifically the preferential utilization of glycolysis by pro-inflammatory lineages versus reliance on FAO by immunosuppressive Tregs—serve as a decisive determinant of their differentiation, functional output, and ultimate impact on disease trajectory (172). Fundamental to immunometabolic understanding is the contrast in energy substrate utilization between T cell subsets (173). During activation within pro-inflammatory contexts, naïve CD4^+^ T cells differentiating into Th1 and Th17 effector lineages undergo a metabolic shift toward aerobic glycolysis and enhanced glutaminolysis, even under normoxic conditions; This metabolic reprogramming, analogous to the Warburg effect, facilitates rapid biomass generation and biosynthetic precursor supply, underpinning proliferation and the secretion of inflammatory cytokines such as IL-17 and IFN-γ (174). Conversely, the differentiation, functional maintenance, and long-term survival of Tregs are intrinsically linked to mitochondrial oxidative phosphorylation (OXPHOS), predominantly fueled by FAO (37, 38). This metabolic phenotype supports a favorable redox balance and is essential for the stable expression of the lineage-defining transcription factor Foxp3, enabling Tregs to persist and function within the lipid-rich, often nutrient-poor, plaque milieu (175, 176). Thus, a microenvironmental or signaling-driven bias favoring glycolysis over FAO can disrupt the Th17/Treg balance, amplifying vascular inflammation (177). The mechanistic target of rapamycin (mTOR) and AMP-activated protein kinase (AMPK) pathways function as central integrators of environmental signals with cellular metabolic programs (178, 179). mTOR complex 1 (mTORC1), activated by nutrient and growth factor signaling, acts as a master promoter of anabolic metabolism, driving glycolysis, lipogenesis, and protein synthesis while suppressing catabolic processes like autophagy (180). Its activity is crucial for the differentiation of Th1 and Th17 cells (181). In atherosclerosis, risk factors like hyperhomocysteinemia can inappropriately activate the phosphatidylinositol 3-kinase (PI3K)/(RAC-alpha serine/threonine-protein kinase)AKT/mTOR pathway in CD4^+^ T cells, leading to a shift toward glycolysis and lipogenesis as elevated homocysteine levels upregulate pyruvate kinase M2 (PKM2) through mTOR signaling, which enhances the production of pro-inflammatory cytokines such asIFN-γ and accelerates early atherosclerotic lesion formation in preclinical models, while the genetic ablation or inhibition of PKM2 in T cells mitigates this inflammatory phenotype and slows disease progression (182). In opposition, AMPK serves as a cellular energy sensor activated under conditions of metabolic stress. It promotes catabolic pathways, including FAO and mitochondrial biogenesis, and inhibits mTORC1 (182). Pharmacological or endogenous activation of AMPK in CD4^+^ T cells fosters Treg differentiation and enhances their suppressive capacity by bolstering oxidative metabolism (182). The dynamic equilibrium between the anabolic mTOR and catabolic AMPK pathways therefore constitutes a fundamental metabolic checkpoint directing T cell fate. Local metabolic byproducts further fine-tune this balance through direct modulation of T cell function and epigenetic-like regulation. Lactate, abundantly generated by glycolytic vascular and immune cells within hypoxic plaques, transcends its role as a metabolic waste product to function as a signaling molecule (183). It can induce “lactylation,” a novel form of post-translational modification on histones and other proteins, thereby influencing gene expression programs (184). In T cells, a lactate-enriched microenvironment may reinforce glycolytic metabolism and stabilize pro-inflammatory phenotypes, potentially promoting Th17 responses while impairing Treg functionality (185). Additionally, the dyslipidemic environment inherent to atherosclerosis directly interfaces with T cell immunometabolism (186). Circulating and plaque-derived fatty acids can be internalized by T cells, altering their metabolic state and functional polarization even prior to antigen encounter (187, 188). For instance, certain saturated fatty acids may predispose T cells toward pro-inflammatory effector programs, while specific polyunsaturated fatty acids could support Treg stability, illustrating a direct link between systemic lipid metabolism and immune cell fate within the vessel wall (189). In addition to the well-characterized glucose and lipid metabolic programs that shape T-cell fate, tryptophan catabolic reprogramming—operating through indoleamine 2,3-dioxygenase-1 (IDO1)-mediated degradation of tryptophan along the kynurenine pathway to promote Treg induction and expansion, while Tregs reciprocally modulate IDO1 enzymatic activity and expression via constitutively secreted cytotoxic T-lymphocyte associated protein-4 (CTLA-4)—establishes a bidirectional regulatory circuit within the vascular wall that consolidates local immune tolerance, thereby curbing inflammatory responses and attenuating the progression of atherosclerosis, as reported by Forteza et al. (122).

Collectively, these immunometabolic reprogramming events—spanning glucose metabolism, lipid metabolism, and amino acid catabolism—operate in concert to govern Th17/Treg dynamics, emerging as a key determinant of atherosclerotic pathogenesis and an actionable target for therapeutic intervention.

### 4.3 Microbial metabolites: mediators of Th17/Treg balance in atherosclerosis pathogenesis

Emerging evidence positions the gut microbiota—the vast community of commensal microorganisms residing in the gastrointestinal tract—as a pivotal environmental factor shaping systemic immunity and metabolic health, with significant implications for atherosclerosis (190, 191). Beyond its local roles, the gut microbiome functions as a virtual endocrine organ, producing a diverse array of metabolites that enter systemic circulation and directly modulate immune cell function and differentiation (192, 193). A critical axis of this influence is the regulation of the balance between pro-inflammatory Th17 and anti-inflammatory Treg cells (194, 195). Dysbiosis, an alteration in the composition and function of the gut microbiota, can skew this equilibrium toward a Th17-dominant pro-inflammatory state, thereby accelerating atherosclerotic plaque initiation and progression (139, 196). Microbiota-derived metabolites, particularly short-chain fatty acids (SCFAs), serve as key systemic messengers linking gut ecology to vascular inflammation (197, 198). SCFAs exert potent immunomodulatory effects both locally and systemically (199). They enhance Treg differentiation and function through several complementary mechanisms: (1) they act as histone deacetylase inhibitors (HDACi), leading to hyperacetylation of the promoter and enhancer regions of the Foxp3 gene, thereby stabilizing its expression;(2) they engage specific G-protein coupled receptors (GPCRs) such as GPR43 and GPR109a on immune cells, triggering anti-inflammatory signaling cascades; and (3) they modulate cellular metabolism, favoring OXPHOS in Tregs (200–202).

The integrity of the gut vascular barrier is a crucial factor in this axis (203). Dysbiosis and a diet high in saturated fats can compromise intestinal barrier function, leading to increased intestinal permeability or “leaky gut” (204). This allows the translocation of microbial products, such as Lipopolysaccharide(LPS), into the portal circulation, triggering a state of chronic, low-grade endotoxemia (205). LPS, via activation of Toll-like receptor 4 (TLR4) signaling on innate immune cells and potentially T cells themselves, potently stimulates the production of pro-inflammatory cytokines that drive Th17 differentiation and inhibit Treg generation (206, 207). This persistent, systemic inflammatory tone directly fuels vascular inflammation and endothelial dysfunction, creating a permissive environment for atherosclerotic plaque development. Experimental studies in murine models provide mechanistic evidence for the gut microbiota’s role in modulating the Th17/Treg balance during atherogenesis. High-fat diet (HFD) exposure in atherosclerotic models demonstrates the detrimental axis. In ApoE-/- mice, HFD induces gut dysbiosis, compromises intestinal barrier integrity, and triggers a systemic inflammatory cascade (196). Critically, analysis of lamina propria lymphocytes reveals a skewed immune profile favoring Th17 expansion alongside Treg suppression; this intestinal imbalance correlates with increased pro-inflammatory leukocyte infiltration into aortic lesions and accelerated plaque development (196). The model establishes that diet-induced microbial disturbance can disrupt peripheral immune homeostasis to promote atherogenesis. Conversely, interventions that promote microbial symbiosis exert protective effects through immunomodulation. Dietary methionine restriction (MR) in atherosclerotic mice reduces plaque buildup, increases intestinal populations of SCFA-producing bacteria, and enhances SCFA availability, which is associated with an anti-inflammatory shift in aortic cytokine profiles characterized by reduced pro-inflammatory signals and improved Treg function (198).

In conclusion, the gut microbiota represents an important environmental factor that modulates the systemic Th17/Treg equilibrium and influences atherosclerosis pathogenesis through metabolite production and modulation of barrier function. A symbiotic, SCFA-rich microbiome promotes a Treg-dominant, anti-inflammatory state that protects against vascular inflammation. Conversely, dysbiosis, characterized by altered metabolite profiles and increased intestinal permeability, drives a Th17-skewed, pro-inflammatory response that accelerates atherosclerosis. This intricate gut-immune-vascular axis reveals novel therapeutic opportunities. Strategies aimed at restoring eubiosis through dietary fiber supplementation, pre/probiotics, or targeted microbial metabolite administration offer promising avenues to therapeutically recalibrate the Th17/Treg balance, presenting a innovative approach to mitigating atherosclerosis progression.

### 4.4 Additional protein signaling pathways modulating Th17/Treg equilibrium in atherosclerosis

Beyond established epigenetic, metabolic and gut microbiota controls, emerging evidence reveals that diverse protein signaling pathways critically regulate the Th17/Treg balance during atherogenesis. While numerous such pathways have been described, we have selected three representative examples—proprotein convertase subtilisin/kexin 6 (PCSK6), high-mobility group box 1 (HMGB1), and tripartite motif 21(TRIM21)—to illustrate the mechanistic diversity of protein-mediated immune modulation. These specific molecules were chosen to exemplify distinct classes of regulatory proteins, including proteases, danger signals, and intracellular E3 ubiquitin ligases, respectively. Collectively, they demonstrate how mechanistically disparate signaling cascades converge to fine-tune immune cell polarization through unique molecular mechanisms, often creating complex feedback loops within the inflammatory microenvironment of the developing plaque.

The involvement of specific proteases is exemplified by members of the PCSK family. Studies demonstrate that genetic ablation of certain PCSK members induces a systemic pro-inflammatory state characterized by elevated plasma levels of Th17-associated cytokines (208, 209). This protease deficiency leads to expanded populations of activated T cells in lymphoid organs and enhanced secretion of inflammatory mediators. Intriguingly, while such genetic manipulations may augment atherosclerotic burden, they can simultaneously promote features of plaque stability through increased collagen deposition, suggesting these proteases regulate a Th17-driven axis with dual effects on plaque development and morphology (210). Extracellular alarmins, such as HMGB1, serve as critical danger signals linking cellular stress to immune dysregulation (211). Clinical analyses consistently show elevated circulating HMGB1 levels in atherosclerotic patients, which correlate inversely with protective Treg populations (212). Mechanistic investigations reveal that HMGB1 directly perturbs CD4^+^ T cell fate decisions by promoting transcriptional programs favoring Th17 differentiation while simultaneously suppressing Treg generation through the induction of apoptosis and downregulation of key transcriptional regulators (213, 214). This dual action disrupts immune homeostasis, creating a pro-inflammatory milieu that accelerates plaque progression. At the intracellular level, the protein TRIM21 functions as a crucial regulatory checkpoint in Th17-mediated inflammation (215). As an E3 ubiquitin ligase, TRIM21 constrains T cell polarization toward the Th17 lineage (216). Experimental evidence from Trim21-deficient murine models demonstrates that loss of TRIM21 leads to enhanced differentiation of naïve CD4^+^ T cells into Th17 cells, resulting in elevated IL-17 expression within atherosclerotic plaques (217). The expansion of Th17 cells is notably associated with significant changes in the metabolism of the plaque’s extracellular matrix, characterized by reduced MMP activity and increased collagen deposition, resulting in plaques in Trim21-deficient models displaying a unique phenotype with larger lesion sizes but enhanced stability of the fibrous cap (217). This seemingly paradoxical observation is explained by the finding that TRIM21 deficiency leads to decreased MMP expression, which limits collagen degradation and thus contributes to increased collagen deposition. Importantly, this does not contradict the destabilizing role of Th17 cells described elsewhere in this review; rather, it highlights that the net outcome of Th17 responses on plaque stability is determined by the balance of multiple effector pathways, including matrix remodeling. When MMP-mediated degradation is attenuated, the pro-inflammatory effects of enhanced Th17 differentiation may be counterbalanced by enhanced matrix preservation, ultimately favoring a more stable plaque phenotype.

**Figure 3:**
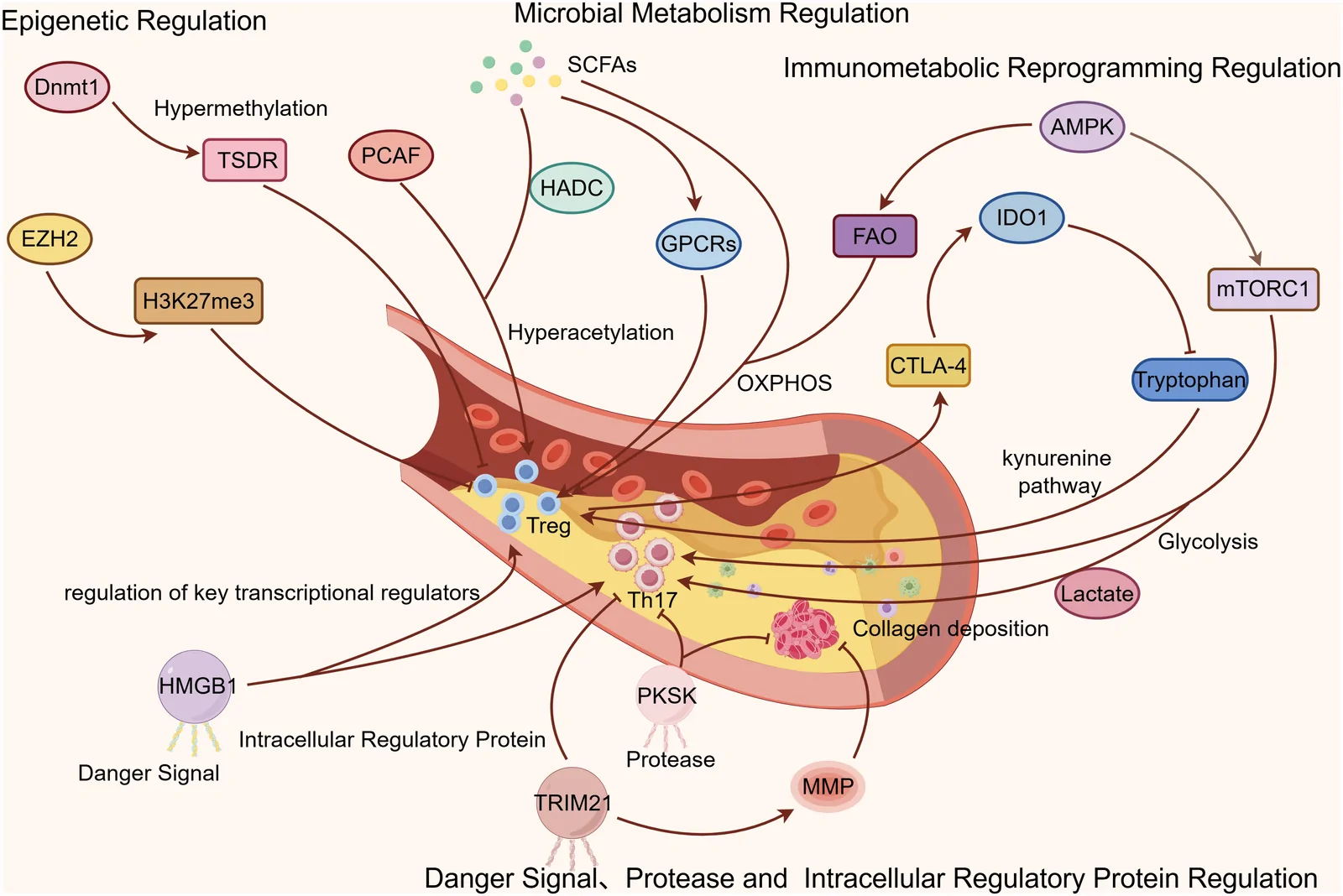
Integrated regulatory network governing Th17/Treg balance in atherosclerosis. The schematic illustrates four regulatory layers—epigenetic regulation, immunometabolic reprogramming, microbial metabolism, and signaling mediators (danger signals, proteases, and intracellular regulatory proteins)—that collectively modulate the differentiation and functional balance of Th17 and Treg cells, thereby influencing the progression of atherosclerotic disease. Epigenetic regulation operates through DNA methylation and histone modifications: Dnmt1 maintains hypermethylation of the TSDR, and EZH2 promotes H3K27me3 stabilization, both of which impair Treg differentiation, whereas PCAF directly acetylates the Foxp3 transcription factor to facilitate Treg commitment. Immunometabolic reprogramming is characterized by opposing metabolic dependencies: AMPK activation, which promotes FAO and suppresses mTORC1, supports Treg differentiation and functional maintenance through FAO-driven OXPHOS, whereas mTORC1-driven glycolysis is required for Th17 cell differentiation. Additionally, IDO1-mediated tryptophan degradation along the kynurenine pathway promotes Treg expansion, while Tregs reciprocally enhance IDO1 enzymatic activity and expression via CTLA-4, forming a bidirectional regulatory circuit within the vascular wall. Microbial metabolism contributes through SCFAs, which expand Treg populations via HDAC inhibition, GPCR targeting, and OXPHOS modulation. Signaling mediators exert context-dependent effects: HMGB1 promotes a Th17-favoring transcriptional program while suppressing Treg generation through apoptosis induction and downregulation of key transcription factors. TRIM21 inhibits Th17 differentiation but enhances MMP activity, reducing collagen deposition and destabilizing plaques. Similarly, PCSK suppresses Th17 differentiation while decreasing collagen deposition, thereby undermining plaque stability. Together, these multilayered regulatory mechanisms orchestrate the Th17/Treg equilibrium, dictating the inflammatory milieu and disease trajectory in atherosclerosis (By Figdraw).

In summary, a sophisticated network of protein signaling pathways—encompassing extracellular proteases, danger signals, and intracellular regulatory proteins—exerts precise control over the Th17/Treg axis in atherosclerosis. These diverse molecular players influence not only the intensity of the inflammatory response but also the qualitative functional output of immune cells, thereby differentially affecting plaque progression, composition, and stability. The continued elucidation of this complex regulatory circuitry expands the potential therapeutic landscape, offering novel immunomodulatory targets for strategies aimed at restoring immune homeostasis and achieving durable plaque stabilization in cardiovascular disease (**Figure 3**).

## 5 Targeting Th17/Treg balance is an important potential target for treating atherosclerosis

### 5.1 Targeting the Th17/Treg balance: natural bioactive compounds as promising therapeutics in atherosclerosis

The therapeutic potential of natural bioactive compounds to correct the Th17/Treg imbalance is gaining significant attention, offering multi-targeted approaches to mitigate atherosclerosis progression. These compounds frequently act via modulating immunometabolic pathways, epigenetic regulation, or the gut-vascular axis, rather than merely lowering lipid levels.

Paeonol (Pae), a phenolic compound derived from “Cortex Moutan”, demonstrates atheroprotective effects in multiple ways (218, 219). Research using hyperlipidemic mouse models shows that the administration of Pae reduces the development of atherosclerotic lesions and lessens vascular fibrosis by modifying gut microbial ecology, specifically by enhancing bacterial populations that produce SCFAs (220). The consequent elevation in SCFA levels drives a systemic rebalancing of adaptive immunity, characterized by an increased splenic frequency of Treg cells and a concomitant decrease in Th17 cells (221, 222). This restored Th17/Treg equilibrium correlates with a favorable shift in the aortic cytokine profile—diminished pro-inflammatory mediators (e.g., IL-1β, IL-6, TNF-α, IL-17) and elevated anti-inflammatory interleukin-10 (IL-10)—and ultimately leads to downregulation of key fibrotic proteins in the vessel wall (220).

Similarly, the polyphenol resveratrol (RES) modulates immune cell function by targeting cellular cholesterol metabolism in antigen-presenting cells (223, 224). In dendritic cells (DCs) derived from the bone marrow of atherosclerotic models, RES enhances cholesterol efflux by upregulating the ATP-binding cassette subfamily A member 1 transporter, which decreases DC activation and their ability to stimulate T cells (225). Consequently, RES treatment *in vivo* not only ameliorates hyperlipidemia and lesion burden but also rectifies the splenic Th17/Treg cytokine imbalance, highlighting a pathway where improving cholesterol homeostasis in innate immune cells indirectly restores adaptive immune balance.

Other compounds directly intercept pro-inflammatory signaling cascades central to Th17 biology. The natural products Sparstolonin B (SSNB) and curcumin (Cur) have been shown to alleviate atherosclerosis by differentially inhibiting the IL-17 receptor A-TAK1-NF-κB pathway, a critical axis for Th17-mediated inflammation (226). While SSNB preferentially targets Toll-like receptor 4 (TLR4)/CD36 to suppress dendritic cell activation and subsequent Th17 differentiation, curcumin exerts stronger effects on inhibiting macrophage inflammation and NF-κB p65 phosphorylation (226). This complementary action underscores how compound combinations can precisely modulate stromal-immune interactions.

Furthermore, inhibition of specific damage-associated molecular patterns can rebalance T cell subsets. Glycyrrhizin (GLY), a direct inhibitor of the alarmin high-mobility group box 1, demonstrates atheroprotective effects in murine models (227, 228). GLY treatment decreases circulating alarmin high-mobility group box 1 levels, positively increasing the Treg/Th17 ratio by enhancing STAT5 activation in Tregs while suppressing STAT3 signaling in Th17 cells, thus promoting a regulatory immune phenotype (229).

In summary, diverse natural compound converge on the pivotal Th17/Treg axis through distinct yet complementary mechanisms. Their ability to modulate the gut microbiome, immunometabolism, specific inflammatory signaling nodes, or damage-associated molecular pattern activity positions them as valuable scaffolds for developing novel, immunologically focused therapeutic strategies against atherosclerosis.

### 5.2 Traditional Chinese medicine formulae in restoring Th17/Treg homeostasis for atherosclerosis management

Beyond single bioactive compounds, multi-component Traditional Chinese Medicine (TCM) formulae represent a holistic therapeutic paradigm that aligns with the complex pathogenesis of atherosclerosis by concurrently modulating multiple pathological networks, including the critical Th17/Treg equilibrium (230, 231). Rooted in a systemic theoretical framework, these formulations demonstrate a capacity for coordinated immunomodulation through multi-target mechanisms, offering a promising complementary approach to conventional cardiometabolic therapy (232, 233).

The therapeutic potential of classical formulae is exemplified by research on the Angong Niuhuang Pill (ANP). Experimental studies using ApoE-/- murine models have demonstrated that administering ANP significantly reduces early-stage atherogenesis caused by a high-fat diet while improving serum lipid levels by favorably modulating the low-density lipoprotein to high-density lipoprotein ratio, altering the systemic inflammatory environment through the reduction of pro-inflammatory cytokines such as IL-6 and increasing anti-inflammatory cytokines like IL-10, decreasing the expression of key inflammatory mediators including IL-1β and TNF-α within the vascular wall, lowering chemotactic signals like CCL2 and its receptor CCR2, reducing matrix-degrading enzymes such as MMP-2 and MMP-9, and supporting these effects with a direct rebalancing of adaptive immunity, as evidenced by the reduced splenic frequency of Th17 cells and downregulation of RORγt, while simultaneously expanding the population of Treg cells and increasing the expression of Foxp3 (234). This restored immune equilibrium correlates with improved plaque stability, evidenced by diminished inflammatory infiltration and preserved collagen architecture in aortic root lesions (234).

Further evidence emerges from studies on the Yangyin Qingre Huoxue Prescription (YQHP), a formula designed according to TCM principles to nourish Yin, clear pathogenic heat, and activate blood circulation. In complex atherosclerosis models involving both hyperlipidemia and an inflammatory trigger, YQHP exhibits comprehensive efficacy. It corrects dyslipidemia, ameliorates systemic inflammation, and favorably adjusts the peripheral Th17/Treg ratio (235). Mechanistically, YQHP appears to precisely target the intracellular signaling hubs governing T cell fate: it suppresses the IL-6/STAT3 axis that promotes Th17 differentiation while potentiating the IL-2/STAT5 pathway essential for Treg development and function (235).

However, it must be acknowledged that the current body of literature specifically investigating the capacity of TCM formulae to target the Th17/Treg axis in atherosclerosis remains limited. While preliminary studies on formulae like ANP and YQHP are promising, they represent only an initial exploration of a vast and complex pharmacopeia. The intricate composition of these formulae, involving synergistic interactions among numerous bioactive constituents, presents both a challenge and an opportunity for research. The mechanisms through which these compound prescriptions achieve systemic immune rebalancing are likely multifaceted and network-based, extending beyond the modulation of single pathways. Therefore, this area warrants significantly deeper and more systematic investigation. Future research should prioritize the elucidation of active component groups, their synergistic mechanisms, and the precise molecular circuits through which they converge to restore Th17/Treg homeostasis. Such efforts will be crucial for transforming the empirical wisdom of TCM into targeted, evidence-based therapeutic strategies for atherosclerosis and other immune-metabolic diseases.

### 5.3 Western pharmacological agents targeting Th17/Treg equilibrium in atherosclerosis

Western pharmacological agents that modulate the Th17/Treg equilibrium can be broadly categorized into two interconnected tiers based on their mechanistic proximity to this axis and their stage of clinical validation. The first tier comprises anti-inflammatory agents that have completed large-scale cardiovascular outcome trials and, while not directly targeting Th17 or Treg cells, act on upstream or broader inflammatory pathways that ultimately influence the systemic inflammatory milieu in which Th17/Treg homeostasis operates. The second tier encompasses emerging pharmacological agents that more directly engage lineage-specific pathways governing Th17 differentiation, Treg stability, or their effector functions, though their clinical efficacy in cardiovascular settings remains to be established. This hierarchical organization allows us to first establish the clinical viability of inflammation-directed therapy in atherosclerosis through completed completed large-scale cardiovascular outcome trials, and then to build upon this foundation by reviewing next-generation agents that promise greater immunological precision. Among the first tier, three agents have generated pivotal human data. The “ Canakinumab Anti-inflammatory Thrombosis Outcomes Study “trial first validated the inflammatory hypothesis of atherothrombosis by demonstrating that canakinumab—a therapeutic monoclonal antibody targeting IL-1β—significantly reduced recurrent cardiovascular events independent of lipid lowering, albeit with an increased incidence of fatal infections compared with placebo (236). Conversely, the “Cardiovascular Inflammation Reduction Trial” found no cardiovascular benefit with low-dose methotrexate, a finding consistent with its failure to suppress IL-1β, IL-6, or C-reactive protein levels, underscoring that pharmacological efficacy depends on engagement of specific, mechanistically-defined inflammatory nodes rather than indiscriminate immunosuppression (237). More recently, Two trials established low-dose colchicine as an effective agent for reducing ischemic events in both post-myocardial infarction and chronic coronary disease populations, positioning this orally available anti-inflammatory drug as a viable adjunct to standard secondary prevention (238, 239). Collectively, these first-tier agents provide three essential lessons that inform the development of more precise Th17/Treg-directed therapies: (1) they offer definitive proof-of-concept that pharmacological modulation of inflammation confers tangible cardiovascular benefit independent of lipid lowering; (2) they demonstrate that therapeutic success requires engagement of specific inflammatory pathways, whereas broad or ineffective target engagement yields no clinical benefit; and (3) their safety profiles—particularly infection risk and signals of non-cardiovascular mortality—underscore the imperative for developing interventions with greater immunological precision to minimize off-target effects.

Building upon this clinical foundation, the second tier of Western pharmacological agents comprises those that more directly interfere with the molecular pathways governing Th17/Treg differentiation, plasticity, and effector function. These agents, often repurposed from other therapeutic areas, exert atheroprotective effects by targeting key immunometabolic signaling nodes, influencing the gut-immune axis, or directly modulating cytokine-driven T cell differentiation.

The thiazolidinedione drug pioglitazone (PIO), a peroxisome proliferator-activated receptor gamma (PPARγ) agonist used in type 2 diabetes management, exemplifies a metabolic modulator with direct immunoregulatory benefits (240, 241). The atheroprotective action of PIO is characterized not by reducing overall plaque volume but by fundamentally altering plaque phenotype through an AMPK-dependent pathway that recalibrates the Th17/Treg equilibrium (9). While PIO administration in hyperlipidemic murine models does not significantly diminish total atherosclerotic lesion area, it stabilizes plaques by enhancing collagen deposition within the lesion core—a structural benefit mechanistically linked to its direct immunomodulatory effect of suppressing pro-inflammatory Th17 cell differentiation while promoting immunosuppressive Treg cell expansion, a dual action entirely contingent upon AMPK activation as evidenced by the complete reversal of these phenotypic changes upon AMPK inhibition (9). This immunometabolic reprogramming, corroborated *in vivo* by a distinct splenic profile of reduced IL-17 and elevated Foxp3 expression, demonstrates that PIO’s therapeutic value lies in its capacity to translate the AMPK-mediated restoration of immune balance into enhanced plaque stability, highlighting a novel strategy for cardiovascular intervention.

Other agents target inflammation through different pathways. Rebamipide, a gastroprotective drug with anti-inflammatory properties, shows significant effectiveness in reducing atherosclerosis in ApoE-/- mice by lowering the formation of atherosclerotic lesions and markers of systemic metabolic disorders with oral administration (242). Its protective effect is closely associated with immunomodulation, as evidenced by a decreased splenic population of Th17 cells and a concurrent increase in Treg frequency (242). This rebalancing of the adaptive immune response, alongside the suppression of key pro-inflammatory cytokines, positions rebamipide as a candidate for mitigating inflammation-driven vascular pathology in metabolic diseases.

Furthermore, the modulation of the gut mi crobiota emerges as a novel mechanism of action for classic anti-inflammatory drugs (243). Aspirin, a cornerstone antiplatelet therapy, exhibits atheroprotective effects that extend to reshaping intestinal microbial ecology (244, 245). In atherosclerotic models, aspirin treatment alters gut microbiota composition, notably increasing the proportion of Bacteroidetes and reducing the Firmicutes to Bacteriodetes ratio (139). The microbial remodeling enhances the production of beneficial SCFAs like propionic acid and alters bile acid metabolism by reducing harmful species such as deoxycolic acid, which together are associated with systemic improvements in immune and inflammatory status that can help rebalance the cyclical Treg/Th17 ratio (139). This illustrates how a conventional drug can achieve vascular protection partly through indirect, microbiota-mediated immunomodulation.

In summary, pharmaceuticals such as pioglitazone, rebamipide, and aspirin demonstrate that targeted immunomodulation is a viable and important therapeutic strategy in atherosclerosis. They act through distinct primary mechanisms—activating metabolic sensors (AMPK), directly suppressing inflammation, or remodeling the gut microbiome—yet converge on restoring the critical Th17/Treg equilibrium. This evidence supports the rationale for exploring the immunomodulatory potential of existing pharmacopeia and for designing novel agents that specifically target this pivotal immunological axis to achieve plaque stabilization and reduce cardiovascular risk.

### 5.4 Other emerging modalities for Th17/Treg rebalancing in atherosclerosis

Beyond pharmacological and herbal interventions, emerging therapeutic modalities highlight novel immunological and nutritional strategies to restore the Th17/Treg equilibrium in atherosclerosis. These approaches, including specific immunoglobulins and essential micronutrients, act through precise immunological mechanisms to enforce immune tolerance and suppress pathogenic inflammation.

A promising immunological strategy involves the use of specific natural antibodies. Immunoglobulin M antibodies targeting phosphorylcholine (IgM anti-PC), which are inversely correlated with cardiovascular risk, have demonstrated potent immunoregulatory capacity (246, 247). *In vitro* studies using peripheral blood mononuclear cells from healthy donors, systemic lupus erythematosus patients, and human atherosclerotic plaques demonstrate that IgM anti-PC selectively promotes the differentiation and expansion of Treg cells under specific culture conditions, while simultaneously significantly suppressing the production of pro-inflammatory cytokines, such as IL-17 and TNF-α, from plaque-derived cells (248). Mechanistically, IgM anti-PC appears to exert its effect by interacting with CD40 on dendritic cells, thereby maintaining them in an immature, potentially tolerogenic state that favors the induction of Tregs over Th17 cells (248). This represents a novel, antibody-mediated mechanism to actively skew the adaptive immune response toward tolerance, offering a potential therapeutic avenue for both autoimmune and atherosclerotic inflammation.

Nutritional immunomodulation represents another accessible strategy, with vitamin D (specifically its active form, 1,25(OH)D) serving as a prime example (249, 250). Long recognized for its immunosuppressive properties, vitamin D directly influences T cell lineage commitment (251). Transcriptomic analysis of splenic T cells from hyperlipidemic low-density lipoprotein receptor knockout (Ldlr-/-) mice reveals that *in vitro* treatment with 1,25(OH)D reprograms cellular signaling pathways. It regulates key pathways involved in T cell activation and differentiation, such as JAK-STAT and HIF-1 signaling, leading to a substantial suppression of pro-inflammatory T cell subsets by reducing the population of Th1 cells, strongly inhibiting IL-17 production, and simultaneously encouraging the differentiation of Tregs (252). This coordinated action—simultaneously dampening pathogenic Th1/Th17 responses while bolstering regulatory mechanisms—positions vitamin D supplementation as a viable adjunctive strategy to correct the immune imbalance central to atherogenesis, particularly in individuals with deficiency.

These emerging modalities underscore the expanding therapeutic landscape for immune-targeted atherosclerosis management. The approach of administering tolerogenic antibodies like IgM anti-PC represents a form of passive immunotherapy designed to directly correct the immunological defect. In contrast, vitamin D supplementation exemplifies nutritional immunomodulation, aiming to provide the molecular substrate necessary for intrinsic immune homeostasis. Both strategies, though distinct in form, share the common goal of re-establishing dominant regulatory control over the immune system. Their development highlights a paradigm shift from purely metabolic intervention toward integrated immunometabolic therapy, aiming not only to reduce plaque burden but also to fundamentally resolve the inflammatory drive of atherosclerosis by restoring the Th17/Treg equilibrium. Future research should focus on optimizing delivery systems for therapeutic antibodies and defining precise dosing and status thresholds for micronutrients like vitamin D to maximize their clinical cardioprotective efficacy.

### 5.5 Next-generation precision immunotherapies and translational challenges

Beyond conventional pharmacological and natural product-based approaches, next-generation precision immunotherapies are emerging as transformative strategies for rebalancing the Th17/Treg axis in atherosclerosis. Among these, chimeric antigen receptor-engineered regulatory T cells (CAR-Tregs) represent a particularly promising frontier (253). By redirecting Tregs to recognize disease-relevant antigens, CAR-Tregs enable antigen-specific immunosuppression at sites of vascular inflammation, thereby overcoming the limitations of polyclonal Treg therapy, which suffers from low frequency of disease-relevant clones and limited efficacy in autoimmune contexts (254). Notably, recent preclinical evidence has demonstrated that anti-oxidized low-density lipoprotein (OxLDL) CAR-Tregs effectively attenuate pro-atherogenic foam cell formation *in vitro* and significantly suppress atherosclerotic plaque development in murine models, achieving substantial reductions in lesion burden without compromising systemic immune function (255). The evolution of CAR-Treg platforms—from autologous polyclonal expansion to allogeneic products and universal gene-edited CAR-Tregs—further underscores the rapid pace of innovation in this space (256, 257). The rationale for targeting OxLDL is compelling, as this molecule serves as both a driver of plaque formation and a disease-specific antigen, enabling CAR-Tregs to exert their immunosuppressive functions precisely at sites of pathology while sparing non-diseased tissues (258, 259).

Cell engineering approaches provide complementary strategies for therapeutic applications of functional Tregs generation *in vitro (*260). A noteworthy example is the induction of Tregs from CD4^+^ T cells using the demethylation agent 5-N heterocyclicidine (Aza) (147). By inhibiting Dnmt1, Aza promotes demethylation of TSDR, thereby upregulating Foxp3 expression and driving differentiation of Tregs (261, 262). Translocating Aza-induced Tregs into ApoE^-^/^-^ mice has been shown to expand peripheral Treg populations, attenuate systemic inflammation, and reduce atherosclerotic plaque burden (147). This approach underscores the feasibility of redirecting naïve T cells toward an immunosuppressive Treg phenotype through targeted epigenetic modulation, thereby offering a cell-based therapeutic strategy.

In addition to CAR-Tregs and cell engineering strategies, cytokine-targeted therapies represent a complementary class of interventions with the potential to restore immune equilibrium in atherosclerosis (263). The chemokine CC chemokine ligand 5 (CCL5) has garnered particular attention as a tractable target for preserving Treg-mediated immunosuppression (264, 265). The CCL5–CCR5 axis plays a critical role in recruiting CCR5-expressing Tregs and other regulatory cell populations to dysfunctional endothelium during the asymptomatic phase of atherogenesis (77, 266). Clinical evidence has revealed that Tregs from individuals with dyslipidemia or stable coronary artery disease exhibit elevated CCR5 expression, accompanied by increased circulating levels of CCL5 and other inflammatory mediators (77). Cytokine receptor blockade of CCR5 with D-Ala-peptide T-amide (DAPTA) has been shown to restore Treg frequencies in high-fat diet-induced atherosclerotic mouse models and, in the partial ligation of carotid artery combined with high-fat diet-induced model, to promote plaque remodeling characterized by increased smooth muscle cell content and reduced macrophage accumulation, collectively indicating a shift toward enhanced lesion stability (78). These findings position the CCL5–CCR5 chemokine axis as a promising cytokine-directed strategy for preserving regulatory cell-mediated immunosuppression in the early stages of atherosclerosis, potentially retarding lesion progression before clinical manifestations emerge.

Despite their substantial promise, the clinical translation of these next-generation precision immunotherapies faces formidable challenges that must be critically addressed. Safety concerns represent the foremost barrier: cell-based therapies such as CAR-Tregs and Aza-induced Tregs carry inherent risks including off-target immunosuppression, unintended clonal expansion, and potential severe infections (267–269). Moreover, the long-term safety of engineered Tregs in atherosclerosis emains entirely unexplored. Immune specificity poses an equally daunting challenge. Although CAR-Tregs can be engineered to recognize plaque-enriched antigens such as OxLDL, the heterogeneity of atherosclerotic lesions across different vascular beds and among individual patients raises questions about the optimal antigen target and whether CAR-Tregs activated within plaques remain locally confined or recirculate to exert systemic effects (255). With regard to cytokine-targeted strategies, cytokine and receptor signaling pathways are intimately involved in vascular inflammation as well as in various physiological processes, including myocardial repair and host defense (270–272). Consequently, systemic receptor blockade or activation carries the inherent risk of impairing these beneficial functions while attempting to suppress pathogenic inflammatory responses. Clinical feasibility presents additional translational bottlenecks: the manufacturing of autologous CAR-Tregs and Aza-iTregs requires good manufacturing practice (GMP)-compliant facilities, highly skilled personnel, and substantial financial investment, with production costs and timelines that are prohibitively expensive and impractical for a chronic disease affecting millions of patients worldwide (257); the inherent batch-to-batch variability of autologous products complicates standardization, while allogeneic alternatives risk graft-versus-host disease or immune rejection, necessitating additional engineering steps that further increase complexity and cost (273–275).

An additional and often underestimated limitation pertains to the inherent differences between murine models and human atherosclerosis, which collectively constrain the predictive validity of preclinical findings (276, 277). The immunological landscape of murine and human atherosclerosis diverges substantially: mice lack key components of the human immune system, including certain chemokine receptors and adhesion molecules critical for T-cell trafficking, and the composition of their T-cell subsets—particularly the relative abundance and functional plasticity of Th17 and Treg populations—differs markedly from that of humans (278, 279). Moreover, murine models typically rely on genetic modifications such as ApoE^-^/^-^ or Ldlr-/-, which, while enabling rapid lesion development, do not recapitulate the chronic, multifactorial nature of human atherogenesis that unfolds over decades under diverse risk factors including hypertension, diabetes, and smoking (280, 281). Substantial interspecies differences in drug metabolism and pharmacokinetics mean that dosing regimens optimized in mice may not directly translate to effective human therapy (282). Furthermore, the timing of therapeutic intervention in murine studies—often initiated simultaneously with or shortly after diet commencement—does not reflect the clinical reality of treating patients with established, advanced atherosclerosis. Finally, the composition of the gut microbiome, which profoundly influences Treg differentiation and function, varies considerably between mice and humans, potentially affecting the efficacy of immunotherapies targeting the Th17/Treg balance when translated across species (283, 284). Collectively, these interspecies disparities necessitate cautious interpretation of murine findings and support the incorporation of complementary translational models, including ex vivo human tissue assays and non-human primate studies, to enhance the predictive accuracy of preclinical evaluations prior to clinical translation.

Addressing these translational challenges—through the development of universal platforms, integration of safety “suicide switches,” identification of predictive biomarkers, refinement of dosing regimens, and establishment of robust large-animal models—is not merely a regulatory hurdle but a scientific imperative to ensure that the remarkable potential of precision immunotherapies can ultimately be harnessed to benefit patients with atherosclerosis (**Table 1**).

**Table 1:** Summary of potential targeted Th17/Treg balance therapeutics for AS.

Category	Drug name	Model	Mechanism of action	Evidence level	Refs
Natural bioactive compounds	Pae	ApoE-/- mice	IL-1β↓, TNF-α↓, IL-6↓, IL-17↓, Th17↓, Treg↑, IL-10↑	Mice	(220)
	RES	ApoE-/- mice	IL-6↓, IL-17↓, IL-2↑, IL-10↑	Mice	(225)
		Stimulated BMDCs cocultured with the T cells	IL-22↓, IL-17↓, activatived T cells ratio↓, IL-10↑, TGF-β↑	Cell	
	SSNB/Cur	ApoE-/- mice	IL-17↓, Th17↓	Mice	(226)
		Stimulated BMDCs cocultured with the T cells	IL-23↓, TNF-α↓, IL-6↓, Th17↓, TGF-β↑, Treg↑	Cell	
	GLY	ApoE-/- mice	IL-6↓, IL-17↓, Th17↓, IL-2↑, IL-10↑, Treg↑	Mice	(229)
TCM	ANP	ApoE-/- mice	IL-1β↓, TNF-α↓, IL-6↓, IL-17↓, Th17↓, RORγt↓, Treg↑, IL-10↑, Foxp3↑	Mice	(234)
	YQHP	ApoE-/- mice	IL-22↓, IL-6↓, IL-17↓, Th17↓, IL-2↑, IL-10↑, TGF-β↑, Treg↑	Mice	(235)
Western pharmacological agents	PIO	ApoE-/- mice	Th17↓, IL-17↓, Treg↑, Foxp3↑	Mice	(9)
		Stimulated splenocytes	Th17↓, IL-17↓, Treg↑, Foxp3↑	Cell	
	Rebamipide	ApoE-/- mice	Th17↓, IL-17↓, Treg↑, Foxp3↑	Mice	(242)
	Aspirin	ApoE-/- mice	IL-1β↓, TNF-α↓, IL-6↓, IL-17↓, Th17↓, Treg↑, IL-10↑	Mice	(139)
Other emerging modalities	IgM anti-PC	Mononuclear leukocytes isolated from symptom-giving human atherosclerotic plaques	TNF-α↓, IL-17↓, Treg↑	Human	(248)
	1,25(OH)D	Ldlr-/- mice	Th17↓, IL-17↓, Treg↑, IL-2↑, IL-10↑	Mice	(252)
Next-generation precision immunotherapies	CAR-Tregs	Ldlr-/- mice	plaque area↓, foam cell↓, collagen content surrounding plaques↑	Mice	(255)
	Aza-induced Tregs	ApoE-/- mice	plaque area↓, IL-1β↓, IFN-γ↓, collagen content surrounding plaques↑, peripheral Treg↑, IL-10↑, TGF-β↑	Mice	(147)
	DAPTA	High-fat diet-induced mice	Treg↑	Mice	(78)
		Partial ligation of carotid artery combined with high-fat diet-induced mice	macrophage↓, smooth muscle cell↑		

## 6 Conclusions and future perspectives

The Th17/Treg equilibrium represents a central organizing principle in the immunopathogenesis of atherosclerosis. This review establishes that the balance between these functionally antagonistic T cell subsets is not merely a descriptive feature but a dynamic regulatory system whose disruption fundamentally drives disease progression. A Th17-dominant state creates a pro-inflammatory milieu that accelerates plaque development and vulnerability, while Treg predominance promotes vascular homeostasis and stability. The decisive role of this axis positions it as a critical target for therapeutic intervention.

The regulation of this equilibrium operates through multiple, interconnected biological layers. Epigenetic modifications, cellular metabolic states, host-microbiome interactions, and specific protein signaling pathways collectively form a cohesive regulatory network that determines T cell differentiation, plasticity, and function within the vascular microenvironment. This multi-layered control underscores the biological complexity of achieving durable immune rebalancing and suggests that effective therapeutic strategies may need to address this system as an integrated whole rather than through isolated pathway modulation.

Future advancements in this field will depend on addressing several pivotal challenges. Key priorities include delineating the hierarchical relationships between different regulatory mechanisms, defining the temporal evolution of Th17/Treg imbalance during disease progression, overcoming translational hurdles related to safety, immune specificity, and clinical feasibility, and establishing clinically relevant biomarkers that accurately reflect the functional state of this immune axis. Furthermore, developing therapeutic approaches that can simultaneously engage multiple regulatory nodes to achieve synergistic immunomodulation will be essential for translating this knowledge into effective clinical interventions.

In summary, the Th17/Treg axis constitutes a fundamental mechanism in atherosclerosis pathogenesis whose therapeutic manipulation offers a promising strategy for achieving immune homeostasis. Advancing this field requires an evolutionary progression in both research and clinical development: shifting focus from observational correlation toward mechanistic causality, and from interventions targeting isolated pathways toward system-level strategies that respect the integrated nature of immune regulation. Such progression is essential for translating immunomodulation from experimental promise into clinical reality within cardiovascular medicine.

## Glossary

Treg: regulatory T cells
Th17: T helper 17 cells
IL-10: interleukin-10
TGF-β: transforming growth factor-beta
IL-35: interleukin-35
RORγt: Retinoic acid-related orphan receptor gamma t
IL-6: interleukin-6
IL-21: interleukin-21
Foxp3: forkhead box P3;IL-23, interleukin-23
GM-CSF: granulocyte-macrophage colony-stimulating factor
TNF-α: tumor necrosis factor-α
CTLA-4: cytotoxic T-lymphocyte-associated protein 4
CCR5: C-C chemokine receptor 5
CXCR3: C-X-C motif chemokine receptor 3
IL-2: interleukin-2
TSDR: Treg-specific demethylated region
ox-LDL: oxidized low-density lipoprotein
IL-17RA: IL-17 receptor A
ECs: endothelial cells
APC: antigen-presenting cell
VSMCs: vascular smooth muscle cells
CXCL1: C-X-C motif chemokine ligand-1
CXCL8: C-X-C motif chemokine ligand-8
MMPs: matrix metalloproteinases
ROS: reactive oxygen species;PRC2, Polycomb Repressive Complex 2
EZH2: Enhancer of Zeste Homolog 2
H3K27me3: trimethylation of histone H3 at lysine 27
IL-4: interleukin-4
iNKT: invariant Natural Killer T cell
PLZF: Promyelocytic Leukemia Zinc Finger
Dnmt1: DNA methyltransferase 1
Aza: 5-azacytidine
IFN-γ: interferon-gamma
OXPHOS: oxidative phosphorylation
AMPK: AMP-activated protein kinase
mTORC1: mTOR complex 1
IDO1: indoleamine 2,3-dioxygenase-1
CTLA-4: cytotoxic T-lymphocyte associated protein-4
PI3K: phosphatidylinositol 3-kinase
PKM2: pyruvate kinase M2
SCFAs: short-chain fatty acids
HDACi: histone deacetylase inhibitors
GPCRs: G-protein coupled receptors
LPS: Lipopolysaccharide
TLR4: Toll-like receptor 4
HMGB1: high-mobility group box 1
TRIM: tripartite motif
Pae: Paeonol
RES: polyphenol resveratrol
DCs: dendritic cells
SSNB: Sparstolonin B
Cur: curcumin
GLY: Glycyrrhizin
TCM: Traditional Chinese Medicine
ANP: Angong Niuhuang Pill
YQHP: Yangyin Qingre Huoxue Prescription
PIO: The thiazolidinedione drug pioglitazone
PPARγ: peroxisome proliferator-activated receptor gamma
IgM anti-PC: Immunoglobulin M antibodies targeting phosphorylcholine
Ldlr-/-: low-density lipoprotein receptor knockout
CAR-Tregs: chimeric antigen receptor-engineered regulatory T cells
OxLDL: oxidized low-density lipoprotein
Aza: agent 5-N heterocyclicidine
CCL5: CC chemokine ligand 5
DAPTA: D-Ala-peptide T-amide
